# Noninvasive Measurement of Lung Function Using the Inspired Sinewave Technique in Mechanically Ventilated Patients With Acute Brain Injury: A Feasibility Study

**DOI:** 10.1213/ANE.0000000000007456

**Published:** 2025-02-19

**Authors:** Arun G. P. Joseph, Minh C. Tran, Louise Rose, Jaideep J. Pandit, Andrew D. Farmery

**Affiliations:** From the ^1^Nuffield Department of Clinical Neurosciences, University of Oxford, Level 6, West Wing, John Radcliffe Hospital, Oxford, UK; 2 Digital Health & Applied Tech Assessment, King’s College London, Faculty of Nursing, Midwifery & Palliative Care, James Clerk Maxwell Building, London, UK; 3 Nuffield Department of Anaesthetics, Oxford University Hospitals NHS Foundation Trust, Oxford, UK.

Critically ill adults are frequently dependent on invasive mechanical ventilation (IMV) for airway protection and adequate gas exchange.^1,2^ Although ventilatory parameters such as airway pressure and tidal volume are routinely used to monitor mechanical ventilation, more advanced cardiopulmonary metrics such as dead space and pulmonary blood flow are less accessible. The Inspired Sinewave Technique (IST) is a relatively noninvasive approach for measuring these indices.^3–6^ A small and sinusoidally varying amount of N_2_O is added to the gas mixture inhaled by the patient. By comparing the amplitude and phase of the inspired and expired N_2_O concentrations, the IST is able to estimate dead space volume (*V*_*D*_), effective lung volume (ELV), pulmonary capillary blood flow (Qp), and ventilatory heterogeneity (HI).

Such a method requires synchronous dosing of the sinusoidal N_2_O input with inspiration, which may not be possible in patients with ventilator dyssynchrony or irregular breathing patterns. To test the performance of the IST in patients with abnormal breathing patterns, we examined the feasibility of IST in patients with acute brain injury (ABI). Such patients have a higher likelihood of abnormal breathing.^7^

## METHODS

After ethical approval and informed written consent from personal consultees of the patients (see Supplemental Digital Content S1, http://links.lww.com/AA/F228 for ethical issues), we included patients with ABI aged ≥18 years requiring IMV and excluded patients requiring >80% inspired oxygen, unstable intracranial pressures (ICP ≥ 20 mm Hg for >10 minutes),^8^ or spinal cord injury.

The Inspiwave system consists of a mass flow controller and an integrated mainstream infrared gas sensor and flowmeter (Figure A), connected in series with the endotracheal tube. A detailed description of Inspiwave operation has been previously published^3^ (see Supplemental Digital Content S2, http://links.lww.com/AA/F228 for detailed methods). Briefly, at the start of each inspiration, the mass flow controller injected a precise amount of N_2_O into the inspired gas which varied sinusoidally over consecutive breaths (Figure B). The integrated sensor then captured the exhaled airflow, CO_2_, and N_2_O concentrations at a sampling rate of 1000 Hz. For both controlled and spontaneous ventilatory modes, each breath was visually examined noting the expired “nitrogram” (N_2_O equivalent of a capnogram) for signal quality. Similarly, the envelope of the end-tidal N_2_O concentration (Figure B) was examined to determine whether a sinusoidal function could be recovered (MATLAB software for signal processing).

**Figure.**
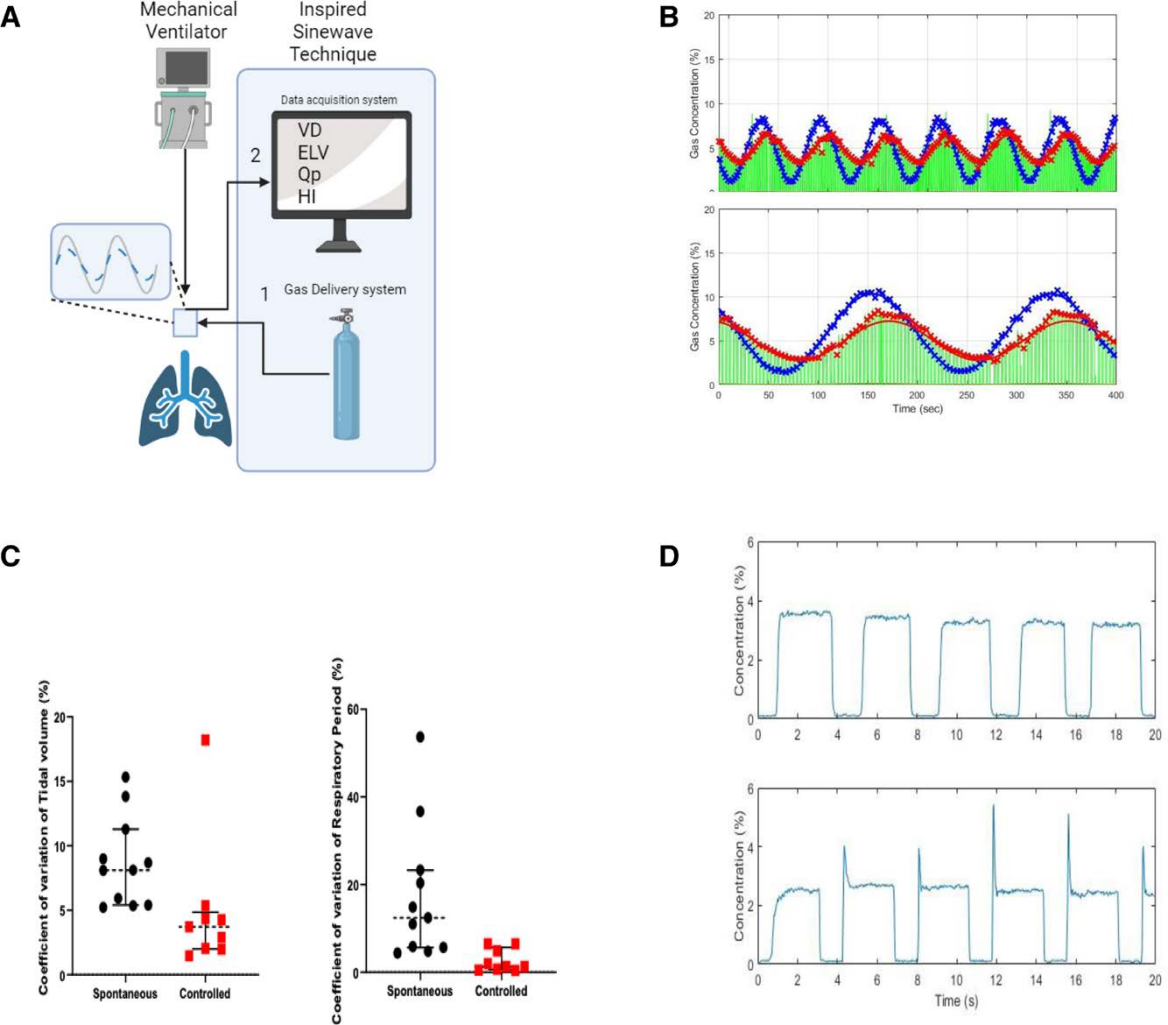
Schematic of the delivery system, examples of measured expirograms, and indices of the stability of breathing patterns. A, (1) Setup of Inspiwave equipment with invasive mechanical ventilation/respiratory support and (2) output of parameters, including the VD, ELV, Qp, and HI. B, End-tidal (green X), inspired (blue X), and expired (red X) N_2_O concentration at 60 s (top) and 180 s (bottom). Continuous red line shows end-tidal points best-fitted to a sine function. Dose was varied sinusoidally over consecutive breath, with a mean concentration of 5% and an amplitude of 4%. The period of sinewave was 180, 90, and 60 s, each lasting 20 min. C, Coefficient of variations (standard deviation divided by the mean for each patient) of tidal volume and respiratory period between controlled ventilation and spontaneous mode of ventilation. The wide range of coefficients in the spontaneously breathing patients reflects irregular breathing in this group. D, Comparison of high-quality (top) and inferior-quality (bottom) waveform patterns. In the bottom plot, there was a delay in the delivery of N_2_O during the first breath, leading to spikes in the N_2_O concentration on expiration in subsequent breaths. ELV indicates effective lung volume; HI, index of ventilator heterogeneity; Qp, pulmonary blood flow rate; VD, ventilatory dead space.

Our feasibility end points were to confirm ability to (a) synchronize tracer dose delivery with early inspiration, regardless of any irregularities of respiration (spontaneous breathing) indicated by intrasubject variance in respiratory period and tidal volumes; (b) obtain sufficient signal: noise ratio to enable measurement of ELV, *V*_D_, Qp, and HI for robust inversion of the mathematical model; and (c) provide reassurance that there are no immediate risks with using IST.

## RESULTS

In total, 20 patients were enrolled in this study, 11 were studied during spontaneous ventilatory modes and 9 during controlled ventilation. The Table shows patient demographics and clinical characteristics. In all patients, we obtained continuous data for 20 minutes with a sufficient signal-to-noise ratio to measure lung function variables (Table and Figure B). We observed no adverse events related to IST. Figure C shows the median coefficient of variation (CoE) of tidal volume and respiratory period in both controlled (n = 9) and spontaneous modes of ventilation (n = 11). In patients in spontaneous mode, the median CoE for respiratory period was 12.4%, and for tidal volume was 8.1% indicating the degree of variance and irregularity of respiration. In contrast, the median CoE values in controlled mode were 1.3% (respiratory period) and 3.7% (tidal volume).

**Table.** Results of Patient Characteristics, Ventilatory Parameters, Vital Signs, Clinical Characteristics, and Inspiwave Output.

Parameter	Mean ± SD	Min	Max
Age (y)	53 ± 13	29	70
Weight (kg)	76 ± 14	50	100
Height (cm)	168 ± 8	155	190
BMI (kg/m^2^)	27 ± 4.3	18.7	38.6
PEEP (cmH_2_O)	6.3 ± 0.3	4.7	10
Ppeak (cmH_2_O)	15.6 ± 5.0	8.0	26.0
Tidal volume (ml)	488 ± 95	352	704
FiO2	0.34 ± 0.10	0.21	0.60
RR (breaths/min)	18 ± 3.8	12	26
HR (beats/min)	83 ± 17	53	115
Other clinical characteristics			%
Days on ventilator	>5 d		40
Mode of ventilation	Spontaneous mode		55
Type of brain injury	Spontaneous acute intracranial hemorrhage		55
	Brain mass lesions		20
	Traumatic acute intracranial hemorrhage		25
Extubation outcomes	Direct tracheostomy		15
	Extubation success		55
	Extubation failure		10
	Terminal extubation		20
Inspiwave output	Median	Min	Max
Deadspace volume—*V*_*D*_ (ml)	118	94	169
Effective lung volume—ELV (l)	1.12	0.5	3.5
Pulmonary blood flow—Qp (l/min)	5.1	1.8	8.5
Ventilatory heterogeneity index—HI (ELV_60_/ELV_180_)	0.92	0.65	1.00

Values include mean, standard deviation, minimum and maximum values for patient characteristics, ventilatory parameters, and vital signs. Other clinical characteristics are expressed as percentages, and median, minimum and maximum values for Inspiwave output.

Abbreviations: BMI, body mass index; ELV, effective lung volume; FiO2, fraction of inspired oxygen; HI, index of ventilator heterogeneity; HR, heart rate; peak, peak inspiratory airway pressure; PEEP, positive end expiratory pressure; Qp, pulmonary blood flow rate; RR, respiratory rate; SD, standard deviation; *V*_*D*_, ventilatory dead space volume.

Definitions: Extubation failure, reintubation after first planned extubation attempt; Extubation success, No requirement for reintubation after first planned extubation attempt; Direct tracheostomy, Direct tracheostomy without a trial of extubation; Palliative extubation, extubation aimed as part of end-of-life care.

When the N_2_O gas was delivered accurately, the nitrogram was a well-defined square wave without spikes (Figure D). Spikes in early expiration indicated dyssynchrony of tracer injection, in turn, due to the delay in N_2_O delivery during the first breath. Out of 60 signals total, we found 2 such inferior-quality signals which we excluded from the analysis. Overall, missing data due to poor signal quality during an assisted spontaneous mode of ventilation was 2%, that is, 1.4 missed breaths per 100 breaths.

## DISCUSSION

In this feasibility study in 20 mechanically ventilated patients with ABI, we found that IST monitoring of cardiorespiratory parameters was feasible and that no safety concerns were noted despite an increased propensity for irregular breathing.

Although we demonstrated the feasibility and short-term safety of IST measurement in our study, the measurements were performed at varied time points of the patient stay in the intensive care unit (ICU), and the study was purely observational. We were thus not able to correlate lung function measures obtained through IST with other measures. Our previous validation studies in animals suggest reasonable accuracy for determining ELV, VD, Qp, and HI when compared with gold standard measures including sulfur hexafluoride (SF_6_) washout_,_^3,4^ bolus thermodilution,^5^ and computed tomography.^4,6^

We have also demonstrated good agreement between lung volumes measured by IST and whole body plethysmography, when normalized for age in healthy volunteers.^9,10^ However, because the IST relies on synchronizing the N_2_O signal with inspiration, it is possible that abnormal breathing patterns may affect the accuracy of the method.

Our validation study of *V*_*D*_ measured by IST and with SF6 wash-in or washout in animals indicated agreement between the 2 measurements.^3^ The ELV estimated by the IST at the bedside also correlated well with measurements by computed tomography and the SF_6_, with concordance rates >98%.^4^ These findings support the potential use of the IST to monitor bedside trends in lung volume.

Key questions for future studies include (i) associations between IST measurements and clinical outcomes; (ii) the potential role of IST measurements in selecting optimal ventilator settings; (iii) the role of IST measurements in weaning outcomes; (iv) the long-term safety of chronic N_2_O exposure; and (v) better understanding of how IST measurements might inform further insights on “brain-lung” cross-talk in ventilated patients with ABI.

## DISCLOSURES

**Conflicts of Interest:** J. J. Pandit is Editor-in-Chief of Anesthesia & Analgesia: he was not involved in the handling of this manuscript. A. D. Farmery is medical advisor to VentDx. No other authors declared Conflicts of Interest. **Funding:** The National Institute for Health Research II-LA-0214- 20005 and NIHR 200029 (2022/23) funded IST development (A.D.F.). From 2023 onwards, the project was funded by a BRC4 Grant (Surgical Innovation and Technology Evaluation). The initial development of the project was through EPSRC funding. **This manuscript was handled by:** Avery Tung, MD, FCCM.

## Supplementary Material


