# Supplementary material for: Noninvasive Measurement of Lung Function Using the Inspired Sinewave Technique in Mechanically Ventilated Patients With Acute Brain Injury: A Feasibility Study
Source: Anesth Analg. 2025 Feb 19;141(3):663–6. doi: 10.1213/ANE.0000000000007456 (PMC12327502; doi:10.1213/ANE.0000000000007456)
Supplement: Supplementary file 1 [file ane-141-663-s001.docx]

**Supplementary material for ethical approval and Inspired sinewave technique**

**Supplement S1: Ethical Approval**

Ethical approval for the study and subsequent amendments was obtained through the Health Research Authority, National Health Services, UK (17/SC/0189), and written informed consent was obtained from the personal consultees of the patients. The study was conducted in the neurocritical care unit at the Oxford University Hospitals NHS Foundation Trust, UK. A Personal Consultee was sought by identifying the next of kin from the clinical notes and discussing with the clinical team who they think appropriate to approach to act as a consultee. A personal Consultee was approached and asked to consider whether, in their opinion, the participant would be prepared to take part in the study. They were given written and verbal versions of the appropriate study information material and had their queries clarified.

**Supplement S2: Inspired sinewave technique methods**

The Inspiwave system consists of a mass flow controller (Alicat Scientific, USA) and an integrated mainstream infrared gas sensor and flow meter. This equipment was connected in series with the patient’s endotracheal tube (ETT). On initial connection, we clamped the ETT to maintain positive end expiratory pressure and avoid derecruitment. The ETT was then unclamped during measurements. At the start of each inspiration, the mass flow controller injected a precise dose of *N_2_O* into the participant’s inspired gas. A volume (dose) of *N_2_O* was injected early in each breath, and the dose was varied sinusoidally over consecutive breaths with a set mean concentration of 5%, an amplitude of 4%, and a period of 180, 90 and 60 seconds for a 20 minute duration in each of three time-periods. The integrated sensor captured the exhaled airflow and CO_2_ and *N_2_O* concentrations at a sampling rate of 1000 Hz. Measurements were made on a controlled mode (n=9) or a spontaneous mode of ventilation (n=11).

Each breath was visually examined and the quality of the expired ‘nitrogram’ (the N_2_O equivalent of a capnogram) signal noted. Similarly, the envelope of the end-tidal N2O concentration was examined to determine whether a sinusoidal function could be recovered. A summary of the inspired sinewave technique have been elaborated in the appendix section of our previous study : Bruce RM, Phan PA, Pacpaco E,et al. The inspired sine-wave technique: A novel method to measure lung volume and ventilatory heterogeneity. *Exp Physiol*. 2018;103:738–747. <https://doi.org/10.1113/EP086867>
